# A Sensor-Aided System for Physical Perfect Control Applications in the Continuous-Time Domain

**DOI:** 10.3390/s23041947

**Published:** 2023-02-09

**Authors:** Paweł Majewski, Wojciech P. Hunek, Dawid Pawuś, Krzysztof Szurpicki, Tomasz Wojtala

**Affiliations:** Faculty of Electrical Engineering, Automatic Control and Informatics, Opole University of Technology, Prószkowska 76 Street, 45-758 Opole, Poland

**Keywords:** continuous-time systems, perfect control, practical implementation, real-life plant, state-space description

## Abstract

The recently introduced continuous-time perfect control algorithm has revealed a great potential in terms of the maximum-speed and maximum-accuracy behaviors. However, the discussed inverse model-originated control strategy is associated with considerable energy consumption, which has exceeded a technological limitation in a number of industrial cases. In order to prevent such an important drawback, several solutions could be considered. Therefore, an innovative perfect control scheme devoted to the multivariable real-life objects is investigated in this paper. Henceforth, the new IMC-related approach, strongly supported by the vital sensor-aided system, can successfully be employed in every real-time engineering task, where the precision of conducted processes plays an important role. Theoretical and practical examples strictly confirm the big implementation potential of the new established method over existing ones. It has been seen that the new perfect control algorithm outperforms the classical control law in the form of LQR (considered in two separate ways), which is clearly manifested by almost all simulation examples. For instance, in the case of the multi-tank system, the performance indices ISE, RT, and MOE for LQR without an integration action have been equal to 2.431, $2.4\times 10^{2}$, and $3.655\times 10^{-6}$, respectively, whilst the respective values 1.638, $1.58\times 10^{2}$, and $1.514\times 10^{-7}$ have been received for the proposed approach.

## 1. Introduction

The paradigm of designing the “ideal” control in modern automation systems has remained the same for decades. Designers and scientists constantly address inconveniences regarding fulfilling exorbitant quality indices related to the accuracy and dynamic properties of the control process. Most approaches focus on obtaining an “optimal” solution, i.e., a consensus between the efficiency and the acceptable energy consumed by the aforementioned system [1,2,3]. This approach leads to many problems in the context of minimizing/maximizing multi-criteria quality indices [4,5,6,7]. Moreover, often the optimization algorithm itself exceeds the computational capabilities of the system on which the target control system is to be implemented in, which opens the possibilities for the artificial intelligence systems (AI) [8,9,10,11,12]. However, an alternative seems to be solutions that, from the set of numerous performance indices, focus only on the one parameter, e.g., accuracy, and regardless of the others, they are designed to keep the system in a given state (even at the expense of high control energy) [9,13,14]. The result mentioned above seems to be trivial, but it can make the plant independent of the entire machinery of optimization research or of implementing the AI. One of the considered approaches is the Continuous-Time Perfect Control (CTPC), which fits into the paradigm of the Inverse Model Control (IMC) systems [15]. The aforementioned perfect algorithm, developed for both discrete- and continuous-time systems, has already been supported by numerous publications, which confirms its significant implementation potential [15,16,17,18]. The significant merit involves obtaining a reference value in an extremely short time. On the other hand, it requires high-energy injection, which means that it may not be feasible for some class of systems, especially those with high inertia. Nevertheless, studies have shown that, in non-square plants, it is possible to reduce the said energy injection even up to 80% in the context of an appropriate choice of so-called degrees of freedom emerging in a properly selected inverse [18]. Moreover, it is possible to select good control parameters for the selected operating point while maintaining low energy consumption and high accuracy [9,15,18,19,20,21,22]. However, the reduction mentioned here, even at the level of ten percent, may not be sufficient for some systems. Therefore, the main goal of this paper is to introduce a new approach that makes it feasible to use the perfect control algorithm in real-life tasks. For this purpose, apart from theoretical considerations, a thermal object and a multi-tank system have been investigated by means of simulations. Furthermore, a real experiment has been carried out on a sensor-aided servomechanism. Let us observe that the preliminary studies have shown a possibility to obtain the inverse model control-related sensor-supported measurement system toward the practical implementation. Henceforth, the perfect control-originated maximum-speed and maximum-accuracy behaviors can outperform the properties of classical control methods according to different performance indices. This merit is effectively presented throughout the manuscript.

Furthermore, the similar structures, connected with the control of the DC motor, can be found in [23,24] or by using the Extended State Observer (ESO) for different objects in [25,26,27]. In these cases, special attention has been paid to the fact that measurement issues play a large role in the context of proper sensor selection or its location [28,29,30,31,32,33,34,35,36,37,38,39]. This article is no different; sensors are important elements which play an important role in the context of the new control law. The encoders used in the research object to measure the speed and position values are the incremental ones with 4096 pulses per rotation. Nevertheless, in the case of using lower resolution sensors, some solutions can be found in [40].

This paper is structured as follows. After presenting the system in Section 2, the preliminary information on the generalized σ-inverse, applied control mechanisms, and systems used in simulation studies are introduced in Section 3. In the Section 4, the new developed perfect control law dedicated to real objects is presented. In Section 5 and Section 6, the simulation studies are conducted, whereas the subsequent Section 6, Section 7, Section 8 and Section 9 discuss the obtained results of the real-life sensor-aided system. This paper is finalized by the conclusions and open problems Section 10, which precedes Appendix A.

## 2. System Representation

The proposed new approach in this paper must be described in state-space representation in the following manner:

(1) $$\left\{\begin{array}{cc} \dot{x}\left(t\right)=Ax\left(t\right)+Bu\left(t\right), & x\left(0\right)=x_{0}\\ y(t)=Cx(t) \end{array}\right.,$$

where appropriate forms $A\in R^{n\times n}$, $B\in R^{n\times n_{u}}$, and $C\in R^{n_{y}\times n}$ are state, input, and output matrices, respectively, while x(t), u(t), and y(t) are the *n*-state, $n_{u}$-input, and $n_{y}$-output vectors, respectively. Furthermore, the mentioned description has an initial condition vector $x_{0}$ in the continuous time t=0.

Moreover, due to the Kalman-oriented control ability properties, the considered system must fulfill the condition $n_{u}\geq n_{y}$, i.e., number of system inputs must be greater or equal to the number of outputs.

## 3. Preliminaries

In this section, some essential knowledge, concerning background of this paper’s consideration, has been presented.

### 3.1. Generalized σ-Inverse

The control law devoted in this article fulfills the IMC paradigm and meets, as mentioned in the previous section, assumptions related to system dimensions. Therefore, in the case of the inverse of the non-square system ($n_{u}>n_{y}$), we have to use generalized inverses. As it has recently been shown in numerous articles, σ-inverse seems to be an excellent solution for this purpose [15,17,41]. For the considered systems, the right invertible approach has the following form [15]:

(2) $$B_{\sigma }^{R}=\beta^{T}{\left(B\beta^{T}\right)}^{-1},$$

while left is as follows:

(3) $$B_{\sigma }^{L}={\left(\beta^{T}B\right)}^{-1}\beta^{T},$$

where β stands for the so-called degrees of freedom matrix.

Introduced Equations (2) and (3) are equivalent, or even better than the Moore–Penrose inverse, in some sense. Moreover, they are the basis for continuous-time perfect control, which is introduced in the next paragraph.

**Remark** **1.** *In spite of the proposed σ-inverse, it is possible to use commonly known Moore–Penrose inverse in perfect control approach. However, this involves limiting the corrective possibilities.*

### 3.2. Continuous-Time Perfect Control

The newly developed Real Continuous-Time Perfect Control (RCTPC) introduced in this article was based on the CTPC mechanism. Therefore, let us remind some important aspects of the recalled approach.

For the continuous-time system given by Equation (1), the control signal of CTPC is defined in the following manner [15]:

(4) $$u\left(t\right)=\left[-{\left(\mathrm{CB}\right)}^{R}\mathrm{CA}-B^{R}M\right]x\left(t\right),$$

where M is defined as:

(5) $$M=C^{R}\frac{1}{dt}\left[Cx\left(t_{k-1}\right)-y_{\mathrm{ref}}\left(t_{k}\right)\right]x^{L}\left(t_{k-1}\right),$$

where ${\left(.\right)}^{R}$ and ${\left(.\right)}^{L}$ stand for every right and left generalized inverse, respectively, whilst the notation given as $y_{\mathrm{ref}}\left(t_{k}\right)$ is a reference value. It should be emphasized that, used in Equation (5), operators $t_{k-1}$ and $t_{k}$ are representation of the continuous-time *t* in the form of $t_{k}=t_{k-1}+dt$ where dt→0.

**Remark** **2.** *Although the CTP control allows for the use of various generalized inverses for non-square matrices, it is suggested to use the previously mentioned σ-inverse, which by implementing the degrees of freedom matrix β makes it possible, e.g., to reduce the energy of the control signal [18].*

The control law presented in Equation (4) can be rapidly verified. For this purpose, let us apply relations between two adjacent continuous-time function values in the form [15]:

(6) $$f\left(t_{k}\right)=f\left(t_{k-1}\right)+\dot{f}\left(t_{k-1}\right)dt,$$

where f(t) stands for any continuous function. After we substitute the mentioned control with relation to Equation (6) into state vector Equation (1), the result takes the following form:

(7) $$x\left(t_{k}\right)=\left(I_{n}+\left(A-B{\left(\mathrm{CB}\right)}^{R}\mathrm{CA}-M\right)dt\right)x\left(t_{k-1}\right).$$

Finally, the target equation of the output, after collecting the all above together, can be proved as follows [15]:

(8) $$\begin{array}{cc} y(t_{k}) & =\left(C-CMdt\right)x\left(t_{k-1}\right)=Cx\left(t_{k-1}\right)-\underset{I_{n_{y}}}{\underset{︸}{CC^{R}}}\frac{1}{dt}dt\\ & \cdot \left[Cx\left(t_{k-1}\right)-y_{\mathrm{ref}}\left(t_{k}\right)\right]\underset{\mathrm{unity}}{\underset{︸}{x^{L}\left(t_{k-1}\right)x\left(t_{k-1}\right)}}\\ & =y_{\mathrm{ref}}\left(t_{k}\right). \end{array}$$

The above consideration confirms the accuracy of the proposed approach. It has been shown that the CTPC can provide high accuracy of control objects after just one simulation time step [15]. Nevertheless, it demands high energy injection of the control signal, which excludes its use in real systems with high inertia. Therefore, the next main section is a solution to address the mentioned inconvenience in case of new RCTPC law. Meanwhile, the next two following subsections introduce well-known control laws used to verify the proposed new approach.

### 3.3. The LQ Regulation

In order to verify and compare the correctness of the new RCTPC application, an approach based on the Linear Quadratic Regulator (LQR) has been considered. Such a mechanism is structurally stable, although an appropriate chosen value of the control energy is very important for its facility and its proper operation. A high energy value allows us to obtain a reference value at the output in a relatively short time. However, technological or financial limitations do not permit the system to achieve infinite (“ideal”) control signal values. In the case of the need to reduce the consumption of control energy, it possible to achieve this by the proper selection of some parameters. In consequence, the reference value is still guaranteed. Nevertheless, the time to reach the set point is lengthened. The main parameters of this regulator are P—positive symmetric and **Q**—positive symmetric semi-definite matrices. The value of the first one is responsible for the input of the system, while the second, for its output. These structures are important in the robustness properties of the control system.

In the basic approach, without integrating action, due to appropriate values of the mentioned matrices, the cost function index is minimized by fulfilling Equation [42,43,44,45]:

(9) $$J=\int_{0}^{T}\left\{{\left[y_{\mathrm{ref}}-y\left(t\right)\right]}^{T}P\left[y_{\mathrm{ref}}-y\left(t\right)\right]+u^{T}\left(t\right)\mathrm{Qu}\left(t\right)\right\},$$

where the used signals result from system Equation (1).

An important issue in this type of control is the feedback from the state vector, not the output of the system, as for many control cases. Moreover, control laws in a great number of studies are presented to achieved zero value. However, in case of any reference value, the second used term should be used in the following manner [45]:

(10) $$u\left(k\right)=-K_{f}x\left(t\right)+K_{r}y_{\mathrm{ref}},$$

where the feedforward matrix $K_{r}$ is the gain inverse matrix for the steady-state system and is defined as follows [45]:

(11) $$K_{r}={\left(B^{T}\mathrm{SB}+R\right)}^{-1}B^{T}{\left[I_{n}{\left(A-{\mathrm{BK}}_{f}\right)}^{T}\right]}^{-1}C^{T},$$

whilst the feedback matrix $K_{f}$ can be obtained from the following Equation [45]:

(12) $$K_{f}={\left(B^{T}\mathrm{SB}+Q\right)}^{-1}B^{T}\mathrm{SA}.$$

The occurred matrix S comes form the Riccati equation, and is a solution to the problem of the LQ Regulator [45]:

(13) $$S=A^{T}\left[S-\mathrm{SB}{\left(B^{T}\mathrm{SB}+Q\right)}^{-1}B^{T}S\right]A+P.$$

The presented control law is well-known and used due to its invaluable possibilities in the control process. Nevertheless, in the next paragraph we consider its variation with integrating action to wide the comparative capabilities for the new introduced RCTPC mechanism.

### 3.4. The LQR with Integrating Action

Another type of control, which has been used to compare with new approaches, is modified LQR. The added aspect is an integrating action which is responsible for the amplification of the output signal and ensures that the reference value is received by minimizing the static error. In a latter part of the paper, the mentioned control has been used according to the following Equation [45]:

(14) $$u\left(t\right)=-K_{f}x\left(t\right)+K_{r}y_{\mathrm{ref}}\left(t\right)+K_{i}\int_{0}^{t}\left[\left(y_{\mathrm{ref}}\left(t\right)-y\left(t\right)\right)\right]dt,$$

where $K_{i}$ stands for integral gain matrix.

The next paragraphs introduce the objects to be used in the further simulation studies.

### 3.5. Systems under Consideration

A total of three different objects have been considered in this paper. Two of them are real objects in the form of nonlinear and linear dynamics equations, while the third one is a real-life plant. Due to the separation of the simulation part from the practical verification, the third system will be examined later in the manuscript.

The dynamics of the first two mentioned systems show in each case the high inertia and limitations concerning the control signals. Therefore, they are ideal for the verification of the new RCTPC law. The objects under consideration are discussed in the separate subsections below.

#### 3.5.1. The Cascade Multi-Tank System

**Figure 1:**
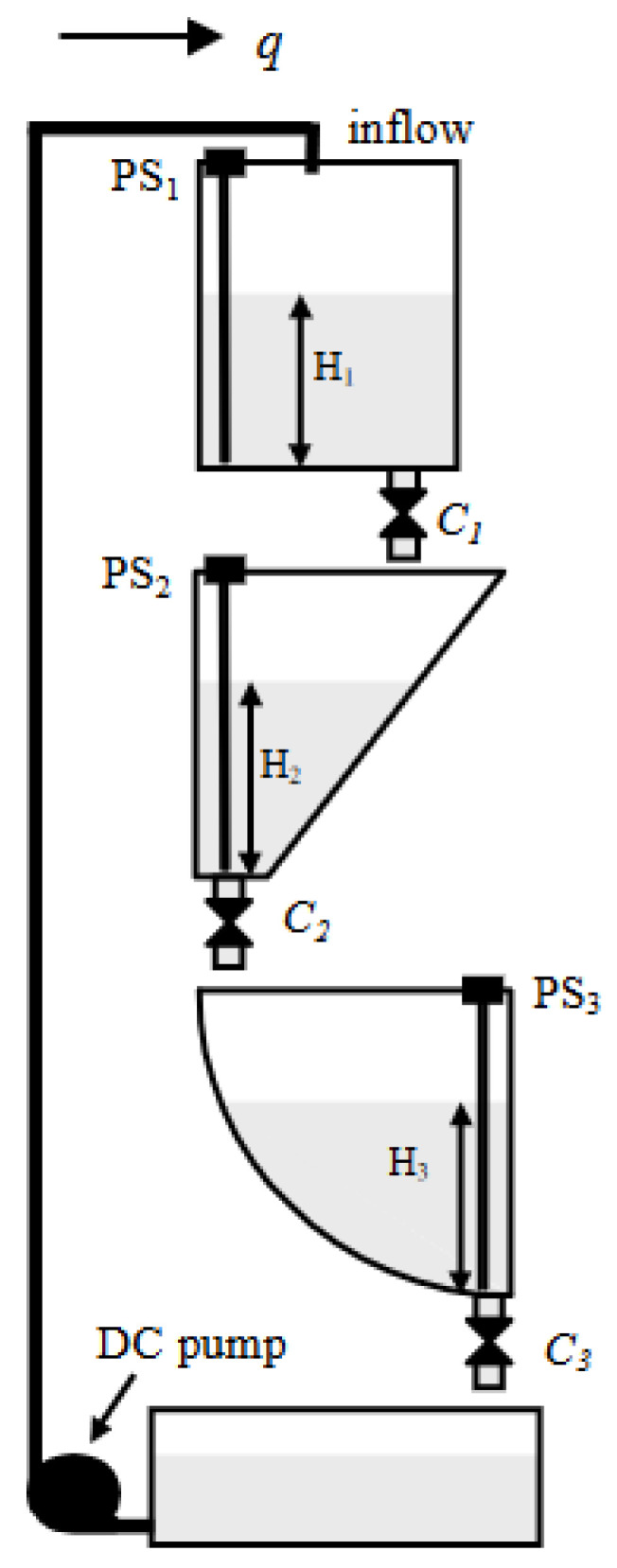
The arrangement scheme of cascade multi-tank system [46].

The first of the analyzed objects is a system of interconnected tanks, developed by the Inteco Company. Its structure is composed of three containers, each with a different configuration (see Figure 1). The purpose of the control process is to stabilize the liquid at an indicated reference value, which can be achieved in three ways: controlling the fill pump by covering the upper tank, controlling the surface throughput of individual valves, or both at the same time (pump and valves) [46,47,48,49,50,51]. The equations of the presented multi-tank system are as follows (based on [47]):

(15) $$\begin{array}{cc} \frac{dH_{1}\left(t\right)}{dt} & =\frac{1}{\alpha_{1}w_{1}}q\left(t\right)-\frac{1}{\alpha_{1}w_{1}}V_{1},\\ \frac{dH_{2}\left(t\right)}{dt} & =\frac{1}{c_{2}w_{2}+\frac{H_{2}\left(t\right)}{H_{2max}}b_{2}w_{2}}V_{1}-V_{2},\\ \frac{dH_{3}\left(t\right)}{dt} & =\frac{1}{w_{3}\sqrt{R^{2}-{\left(R-H_{3}\left(t\right)\right)}^{2}}}V_{2}-V_{3},\\ V_{1} & =C_{1}H_{1}{\left(t\right)}^{\alpha_{1}},\\ V_{2} & =C_{2}H_{2}{\left(t\right)}^{\alpha_{2}},\\ V_{3} & =C_{3}H_{3}{\left(t\right)}^{\alpha_{3}}, \end{array}$$

where symbols and their values’ specification can be found in Table A1 of Appendix A.

#### 3.5.2. The Two-Level Thermal Object

**Figure 2:**
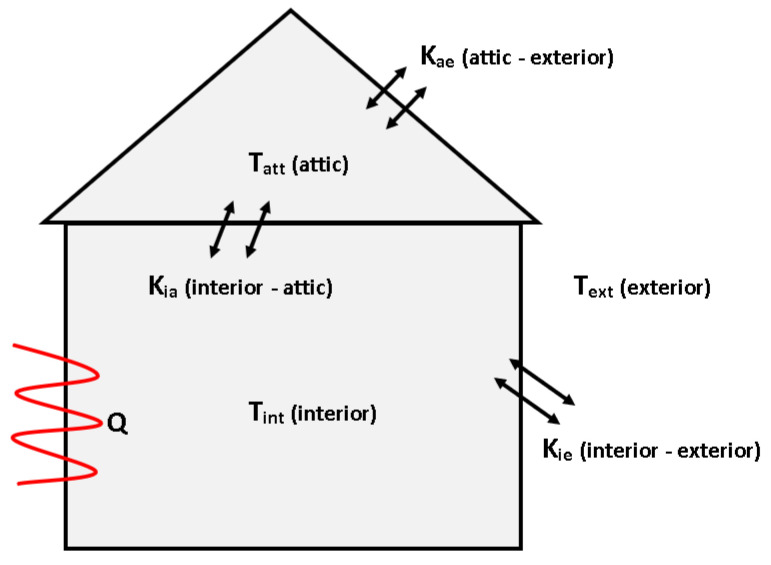
Scheme of the the two-level thermal object (source: authors).

The second system presented in this paper is a thermal object in the form of a house (see Figure 2). The plant consists of two stories of different sizes, heat capacity, and heat loss coefficients between the system elements, the dynamic of which is described by the following differential Equations [52,53,54,55,56]:

(16) $$\begin{array}{cc} \frac{T_{int}\left(t\right)}{dt} & =\frac{\left(Q_{h}-K_{ie}\left(T_{int}-T_{ext}\right)-K_{ia}\left(T_{int}-T_{att}\right)\right)}{C_{vin}},\\ \frac{T_{att}\left(t\right)}{dt} & =\frac{\left(K_{ia}\left(T_{int}-T_{att}\right)-K_{ae}\left(T_{att}-T_{ext}\right)\right)}{C_{vat}}, \end{array}$$

where as before, the symbols and their values can be found in Table A2 of Appendix A.

**Remark** **3.** *Naturally, several types of sensors can be applied to the physical systems. An interesting solution seems to be an involvement of the LK0264A-A-00KQPKG/US and TN-045KCBD18-MFPKG/US tools for measuring both liquid level and temperature, respectively.*

### 3.6. Quality Criteria

The simulation studies of the control of the analyzed objects, as well as practical verification, have been conducted by the application of the following quality indices [9,57,58]:

- ISE—*Integral of Squared Error* defined by
  (17) $$ISE=\int_{t_{0}}^{t}e^{2}\left(t\right)dt,$$
  where e(t) is a control error;
- MOE—*Minimum of energy* which is an integral of squared control signal
  (18) $$J\left(u\right)=\int_{t_{0}}^{t}u^{T}\left(t\right)u\left(t\right)dt;$$
- RT—*Regulation time* which is a time considered from the beginning of the simulation to receiving the tolerance range ±5% of the expected value by the system output.

Two of the presented indices are integral and the third one takes into account the dynamics of the control process. Therefore, they are sufficient to verify the control strategies of the analyzed approaches in this paper.

## 4. The Real Continuous-Time Perfect Control

Despite the significant advantage of the recently introduced perfect control of continuous-time domain systems related to their high accuracy, they possess a serious drawback, which is related to the demand of high energy expenditure [15]. This can be noticed in the control Equation (4), where the term dt→0 is in the denominator (Equation (5)). Therefore, only some particular systems can implement this type of regulation [15]. This paper is an extension of the mentioned approach to the wide class of different real objects, especially with a high inertia property.

The proposed new approach consists of determining the feasibility of the energy injection required in the control process. Certainly, this signal, for an arbitrarily control process, depends on the difference between the initial and the expected value. Nevertheless, within a fixed reference value, it can be limited. One of the approaches to achieve this is the selection of appropriate values of the degrees of freedom in some inverse process (see Remark 2). In some cases, it is possible to reduce the control energy even by 80% according to the following performance index [18]:

(19) $$\begin{array}{cc} J(u,x_{0}) & =\min_{\beta }{\left[\left[-\left(\beta^{T}{\left(\mathrm{CB}\beta^{T}\right)}^{-1}\mathrm{CA}-B^{R}M\right)\right]x\left(t\right)\right]}^{T}\\ & \cdot \left[-\left(\beta^{T}{\left(\mathrm{CB}\beta^{T}\right)}^{-1}\mathrm{CA}-B^{R}M\right)\right]x\left(t\right). \end{array}$$

Unfortunately, not all plants have such high reduction possibilities. Moreover, even the limitation at the mentioned level may also not be a remedy, due to the finite efficiency of the actuator system [18]. Therefore, the approach presented in this paper is to limit the control signal energy at the expense of extending regulation times. Hence, after revealing the restrictions and saturations of the controlled system, the maximum efficiency of the actuators $u_{\max}\left(t\right)$ must be specified. This can allow us to determine the appropriate partial reference value $y_{p.\mathrm{ref}}\left(t\right)$, which finally leads to the total reference value, which will be explained in the following paragraphs.

In order to determine the new control law, taking into account all the above, let us rewrite Equations (4) and (5) into the following statement:

(20) $$\begin{array}{c} \begin{array}{cc} u_{\max}\left(t_{k-1}\right)= & \left[-{\left(\mathrm{CB}\right)}^{R}\mathrm{CA}-B^{R}C^{R}\frac{1}{dt}\left[\mathrm{Cx}\left(t_{k-1}\right)-y_{p.\mathrm{ref}}\left(t_{k}\right)\right]x^{L}\left(t_{k-1}\right)\right]x\left(t_{k-1}\right). \end{array} \end{array}$$

According to relation:

(21) $$\begin{array}{c} B^{R}\cdot C^{R}={\left(\mathrm{CB}\right)}^{R}, \end{array}$$

we can rewrite Equation (20) into the new Real Continuous-Time Perfect Control law:

(22) $$\begin{array}{c} \begin{array}{cc} \; & u_{\max}\left(t_{k-1}\right)\;=\;-{\left(\mathrm{CB}\right)}^{R}\mathrm{CA}x\left(t_{k-1}\right)\;-\;\frac{1}{dt}{\left(\mathrm{CB}\right)}^{R}\mathrm{Cx}\left(t_{k-1}\right)\underset{unity}{\underset{︸}{x^{L}\left(t_{k-1}\right)x\left(t_{k-1}\right)}}\\ \; & +\frac{1}{dt}{\left(\mathrm{CB}\right)}^{R}y_{p.\mathrm{ref}}\left(t_{k}\right)\underset{unity}{\underset{︸}{x^{L}\left(t_{k-1}\right)x\left(t_{k-1}\right)}}=\left[-{\left(\mathrm{CB}\right)}^{R}\mathrm{CA}-\frac{1}{dt}{\left(\mathrm{CB}\right)}^{R}C\right]x\left(t_{k-1}\right)\\ & +\frac{1}{dt}{\left(\mathrm{CB}\right)}^{R}y_{p.\mathrm{ref}}\left(t_{k}\right). \end{array} \end{array}$$

It is easy to prove that the introduced new approach is correct and useful. For this reason, let us transfer the appropriate phrases to the other side and right-multiply them by the expression dtCB. In that case, we receive:

(23) $$\begin{array}{cc} \; & dt\frac{1}{dt}\underset{I_{n_{y}}}{\underset{︸}{\mathrm{CB}{\left(\mathrm{CB}\right)}^{R}}}y_{p.\mathrm{ref}}\left(t_{k}\right)=dt{\mathrm{CBu}}_{\max}\left(t_{k-1}\right)\\ & +\left[dt\underset{I_{n_{y}}}{\underset{︸}{\mathrm{CB}{\left(\mathrm{CB}\right)}^{R}}}\mathrm{CA}+dt\frac{1}{dt}\underset{I_{n_{y}}}{\underset{︸}{\mathrm{CB}{\left(\mathrm{CB}\right)}^{R}}}C\right]x\left(t_{k-1}\right). \end{array}$$

Then, the form of the partial reference value is as follows:

(24) $$\begin{array}{c} y_{p.\mathrm{ref}}\left(t_{k}\right)=dt{\mathrm{CBu}}_{\max}\left(t_{k-1}\right)+\left[dt\mathrm{CA}+C\right]x\left(t_{k-1}\right). \end{array}$$

At the same time, based on Equation (6), the following expression is true:

(25) $$\begin{array}{c} x\left(t_{k}\right)=x\left(t_{k-1}\right)+\dot{x}\left(t_{k-1}\right)dt. \end{array}$$

After taking Equations (1) and (22) into account, we receive the subsequent relation:

(26) $$\begin{array}{cc} \dot{x}\left(t_{k-1}\right) & =\mathrm{Ax}\left(t_{k-1}\right)+B\left[\left[-{\left(\mathrm{CB}\right)}^{R}\mathrm{CA}-\frac{1}{dt}{\left(\mathrm{CB}\right)}^{R}C\right]x\left(t_{k-1}\right)+\frac{1}{dt}{\left(\mathrm{CB}\right)}^{R}y_{p.\mathrm{ref}}\left(t_{k}\right)\right]. \end{array}$$

Now, multiplying Equation (25) by the matrix C, we obtain the output equation according to Equation (1):

(27) $$\begin{array}{c} y\left(t_{k}\right)=\mathrm{Cx}\left(t_{k}\right). \end{array}$$

Ultimately, considering the above Equations (25)–(27), we obtain the following expression:

(28) $$\begin{array}{cc} y(t_{k}) & =\mathrm{Cx}\left(t_{k-1}\right)+C\dot{x}\left(t_{k-1}\right)dt=\mathrm{Cx}\left(t_{k-1}\right)+\mathrm{CAx}\left(t_{k-1}\right)dt-dt\underset{I_{ny}}{\underset{︸}{\mathrm{CB}{\left(\mathrm{CB}\right)}^{R}}}\mathrm{CAx}\left(t_{k-1}\right)\\ & -dt\frac{1}{dt}\underset{I_{ny}}{\underset{︸}{\mathrm{CB}{\left(\mathrm{CB}\right)}^{R}}}\mathrm{Cx}\left(t_{k-1}\right)+dt\frac{1}{dt}\underset{I_{ny}}{\underset{︸}{\mathrm{CB}{\left(\mathrm{CB}\right)}^{R}}}y_{p.\mathrm{ref}}\left(t_{k}\right)=y_{p.\mathrm{ref}}\left(t_{k}\right). \end{array}$$

As can be seen from the above equation, knowing the capabilities of the control system and the behavior of the state variables, it is possible to determine the value of $y_{p.\mathrm{ref}}\left(t\right)$, which the system can receive after one simulation step time equal to dt. Hence, the searched final reference value can be determined by the following relationship:

(29) $$y_{\mathrm{ref}}\left(t\right)=y_{0}+\int_{0}^{t_{s}}\left(y_{p.\mathrm{ref}}\left(t_{k}\right)-y_{p.\mathrm{ref}}\left(t_{k-1}\right)\right)dt,$$

where $y_{0}$ stands for the initial output of the system.

In order to establish that the proposed approach is correct, let us now consider simulation studies in Section 5, in order to finally move onto a sensor-aided plant and to verify the practical operation of the real-life system in Section 7.

## 5. Simulation Studies

In this section, the results of the simulation studies of the new introduced RCTPC algorithm, for the objects presented in the earlier paragraphs (Section 3.5.1 and Section 3.5.2), have been given. Due to verification reasons, other classical control types mentioned before have been also used, to compare the quality of the new approach.

### 5.1. The Cascade Multi-Tank System Control

**Figure 3:**
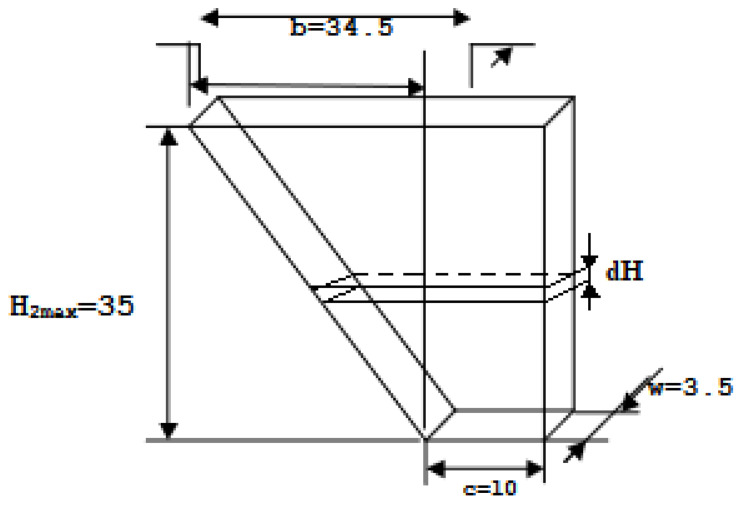
Geometry of the tank in which the water level was maintained—dimensions given in cm (source: authors).

The objective of controlling the system of interconnected tanks was to maintain the liquid level in the middle tank at the reference level equal to $H_{2}$ = 0.25 m (see Figure 3). For this reason, we assumed appropriate parameters represented by C1, C2, C3 (valve settings can be found in Appendix A), whereas pump flow *q* was the variable in the control process. Therefore, depending on the type of control used, different settings were adopted.

In the case of fluid height adjustment using the LQR without an integral action, the following settings have been assumed: R=1 and Q=1.705.

The implementation of the LQR with an integral action received the settings: R= 800, Q= 600, and $K_{i}=10^{-2}$.

For new RCTPC, as in other cases, the initial value of the middle tank was $y_{0}=H_{20}=0$ m. The time step was $dt=1\cdot 10^{-3}$ s and depending on the pump parameters, the partial reference value was assumed at $y_{p.\mathrm{ref}}=10^{-5}$ m.

**Figure 4:**
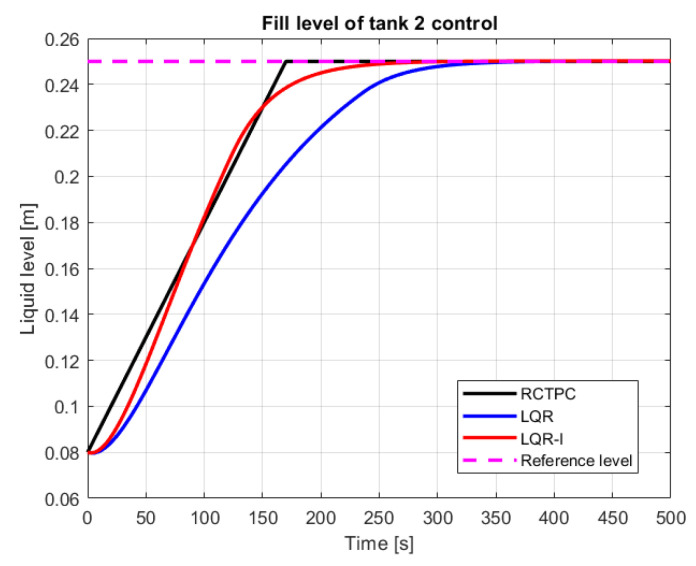
Control process of the filling of the second tank (source: authors).

The simulation studies of stabilization of the liquid height of the second tank have been performed for each of the introduced approaches. Some of the results for the time domain plot are presented in Figure 4, while the used quality criteria are analyzed in the next section.

### 5.2. The Two-Level Thermal Object Control

**Figure 5:**
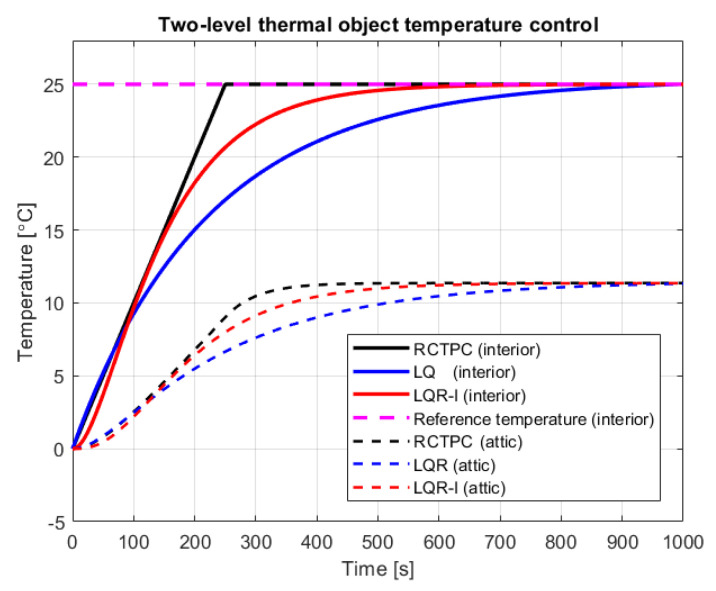
Interior temperature control (source: authors).

The purpose of controlling a two-level thermal object was a obtaining the indoor room temperature at a value of $T_{int}$ = 25 °C. For this reason, the appropriate heat power $Q_{h}$ had to be selected. As it results from the characteristics of the room (see Figure 5), the whole heating process has an effect on the attic temperature, which on the other hand, has an effect on the temperature being regulated in the internal room. For simulation studies, it has been assumed that the temperature of both rooms has an initial value $T_{int0}=T_{att0}$ = 0 °C. Meanwhile, a disturbance of the control process is the external temperature of the system with the value $T_{ext}$ = 0 °C.

In the same manner as for the multi-tank system, simulation studies have been performed for three control strategies including the new introduced one in this paper.

In the case of temperature control using the LQR controller without an integral action, the following parameters have been assumed: $Q=0.889\cdot 10^{-3}$, and $R=3.195\cdot 10^{-7}$.

The LQR control with an integral action has been accomplished with the settings: $Q=6\cdot 10^{1}$, $R=1\cdot 10^{-4}$, and $K_{i}=5\cdot 10^{1}$.

For all types of control, the initial temperature was $y_{0}=T_{int0}$ = 0 °C. Using the new RCTPC algorithm, the partial reference value was raised with the time step $dt=1\cdot 10^{-3}$ s by the gain $y_{p.\mathrm{ref}}$= $1\cdot 10^{-4}$ °C, until the final reference value $y_{\mathrm{ref}}$ = 25 °C was reached.

The results of the simulation studies concerning the temperature control of a two-level thermal object in time domain are presented in Figure 5. Subsequently, other performance indices have been analyzed in the next section.

## 6. Discussion on the Obtained Simulation Results

The received results, through the prism of the values obtained in Table 1 and Table 2, indicate that the RCTPC has great control potential. The innovative real perfect approach was able to address the task of regulation in the best manner. The worst results in terms of the control accuracy have been achieved by the classic LQR control system. This also applies to the control time, where the differences between the classical controller and the controller with an integrating action were significant. As it can be seen, adding an additional integrator to the LQR-based control made it possible to reduce the settling time of the system and its accuracy in relation to the setpoint at the input. However, more energy expenditure was required to drive the system. In conclusion, the used indices confirmed that the innovative real perfect control approach works and outperforms the classical ones. The mentioned algorithm makes it possible to obtain the reference value in a much shorter time and with a smaller error than other controls that have been considered until now. Moreover, as it can be seen in the simulation figures, the derivative of the signal for controlling the system by LQR is greater than the RCTPC control, which means that the new approach has no maximum values. Thanks to this strategy, it is able to provide a margin for signal growth, which minimizes the saturation phenomenon. On the other hand, it also means that it can increase the RCTPC control results by increasing the partial reference value $y_{p.\mathrm{ref}}\left(t\right)$.

**Figure 6:**
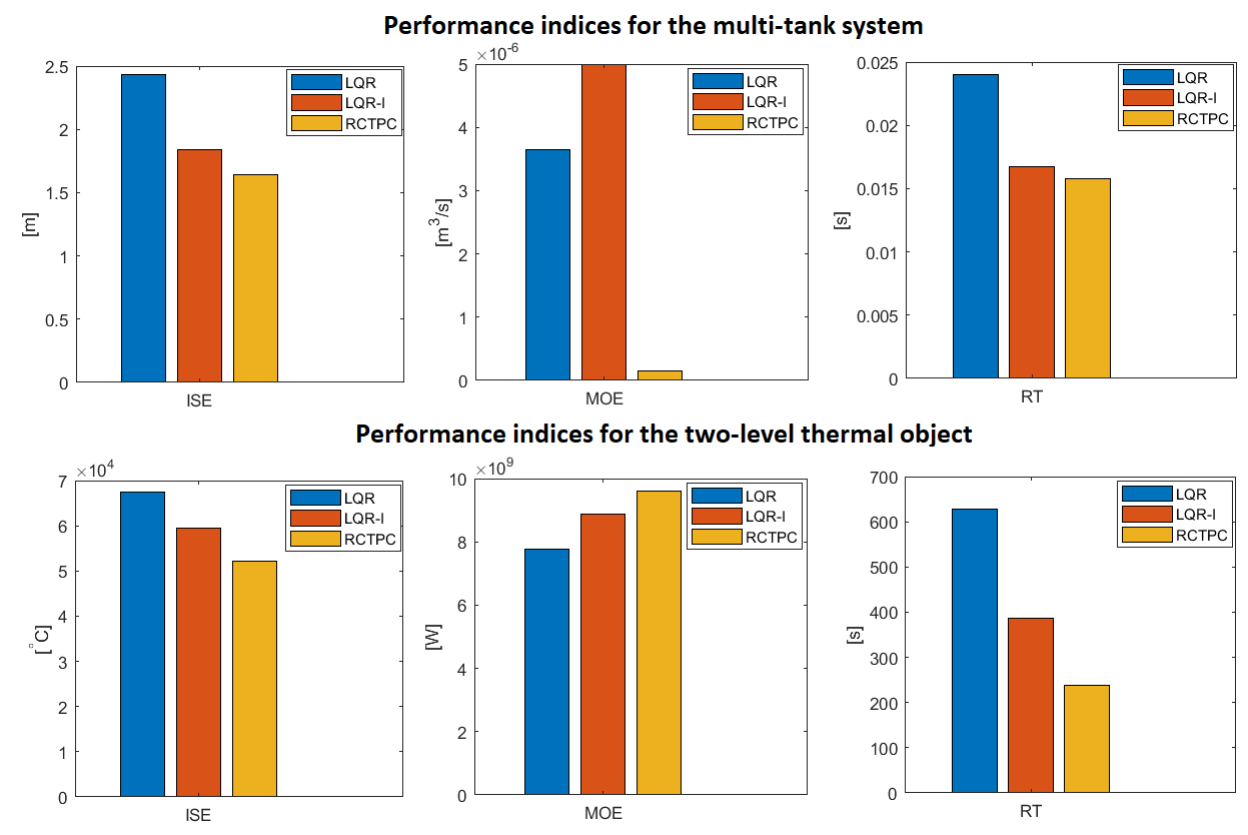
Graphical presentation of Table 1 and Table 2 (source: authors).

Figure 6 seems to better describe the results of Table 1 and Table 2.

At this point, we end the part focused on the simulation studies. The next part of this paper is devoted to the verification of the newly developed algorithm in the real-life systems.

## 7. The Sensor-Aided System—A Real experiment Setup

After a successful verification of the RCTPC strategy in simulation cases of earlier sections, let us switch to a real experiment. For this reason, a sensor-aided system in the form of a servomechanism didactic set has been investigated.

**Figure 7:**
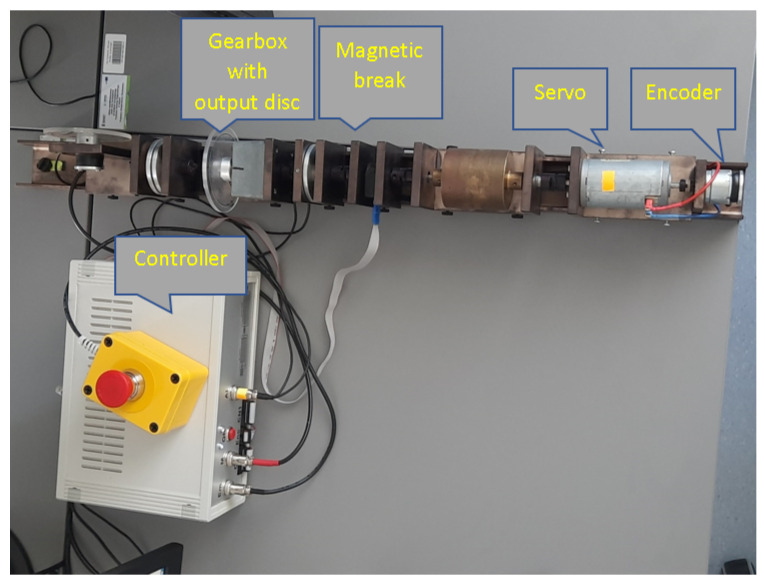
The examined modular sensor-aided servomechanism (source: authors).

The considered device, presented in Figure 7, was connected with a computer to an installed Matlab/Simulink environment. Including a measurement card, such as a structure, gives possibilities to measure the value of the angle and rotational speed, as well as to implement the control algorithm.

**Figure 8:**
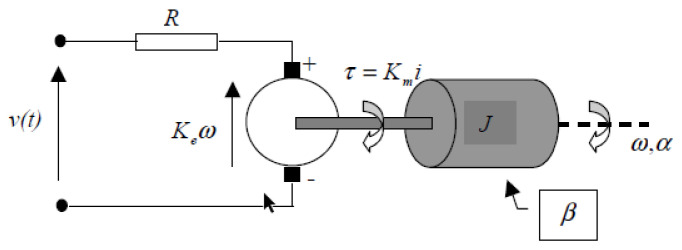
Diagram of DC motor [47].

In the conducted study, a model of the object linearized at the operating point was also used. This strategy was necessary from the point of control view. Therefore, it was necessary to consider two main equations of the DC motor (see Figure 8) describing the electrical (Equation (30)) and mechanical (Equation (31)) parts of the model in the following manner [47]:

(30) $$v\left(t\right)=Ri\left(t\right)+K_{e}\omega \left(t\right),$$

and

(31) $$J\dot{\omega }\left(t\right)=K_{m}i\left(t\right)-\beta \omega \left(t\right),$$

where the parameters used in the model are gathered in Table 3.

Combining the above Equations (Equations (30) and (31)), the subsequent function has been obtained [47]:

(32) $$T_{s}\dot{\omega }\left(t\right)=-\omega \left(t\right)+K_{sm}v\left(t\right),$$

where

(33) $$T_{s}=\frac{RJ}{\beta R+K_{e}K_{m}}\;\;\;and\;\;\;K_{sm}=\frac{K_{m}}{\beta R+K_{e}K_{m}}.$$

Going further, the transfer-function of system angular velocity and angular position are first- and second-order inertia, respectively. This fact can be described in the following manner [47]:

(34) $$G\left(s\right)=\frac{\omega (s)}{u(s)}=\frac{K_{s}}{T_{s}s+1},$$

and

(35) $$G\left(s\right)=\frac{\alpha (s)}{u(s)}=\frac{K_{s}}{s(T_{s}s+1)}.$$

Nevertheless, the new control law introduced in this paper is strictly connected with the state-space description. For this reason, the equations of velocity and angular position (Equations (34) and (35)) can be described by Equation (1), in the form of appropriate matrices, as follows [47]:

(36) $$A=\left[\begin{array}{cc} 0 & 1\\ 0 & -\frac{1}{T_{s}} \end{array}\right],\;B=\left[\begin{array}{c} 0\\ \frac{K_{s}}{T_{s}} \end{array}\right],\;C=I,$$

where I stands for the identity matrix of dimension two.

The rated parameters proposed by the manufacturer are: $v_{max}=12$ [V], $T_{s}=1.04$ [s], and $K_{s}=186$ [rad/s] [47].

After an introduction of a measurement setup, we can now perform research studies in the next section.

## 8. A Real Experiment on a Sensor-Aided Servomechanism

After introduction concerning the configuration of the system, let us focus on an experiment involving a real-life servomechanism. For this reason, many examination procedures have been carried out, mostly with different initial parameters. Nevertheless, in this paper only three of them have been presented.

**Figure 9:**
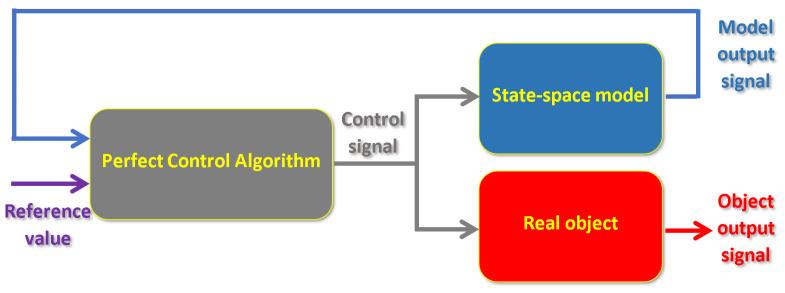
Diagram of control system (source: authors).

For study verification reasons, the authors proposed the structure of the control system shown in Figure 9. This approach significantly simplifies the control plant and also has a lower demand for the computational effort, than in the case of the use of a state observer, e.g., a Luenberger one. Hence, a well-chosen model for a considered system now is crucial. However, the state-space solution of the servomechanism represents rather well the real state of the system and is relatively simple to create. Therefore, for each of the presented experiments, the real-life object has been linearized at a given operating point.

Now, when the configuration of the control process is established, let us finally switch to the research on a real object.

### 8.1. The First Experiment with the RCTPC Law

In the first experiment on a real-life sensor-aided system, the considered reference angle has been assumed at a value of 100°. After a linearization process at a given operating point and the creation of a model of the servomechanism, the following transfer functions have been received (see Equations (34) and (35)):

(37) $$G_{\omega }\left(s\right)=\frac{173.3}{1.04s+1}\;\;\;\mathrm{and}\;\;\;G_{\alpha }\left(s\right)=\frac{173.3}{s(1.04s+1)}.$$

Finally, the expected representation in the examined case, according to Equation (1), is given by the following matrices:

(38) $$A=\left[\begin{array}{cc} 0 & 1\\ 0 & -0.96 \end{array}\right],\;B=\left[\begin{array}{c} 0\\ 14.21 \end{array}\right],\;C=\left[\begin{array}{cc} 1 & 0\\ 0 & 1 \end{array}\right].$$

**Figure 10:**
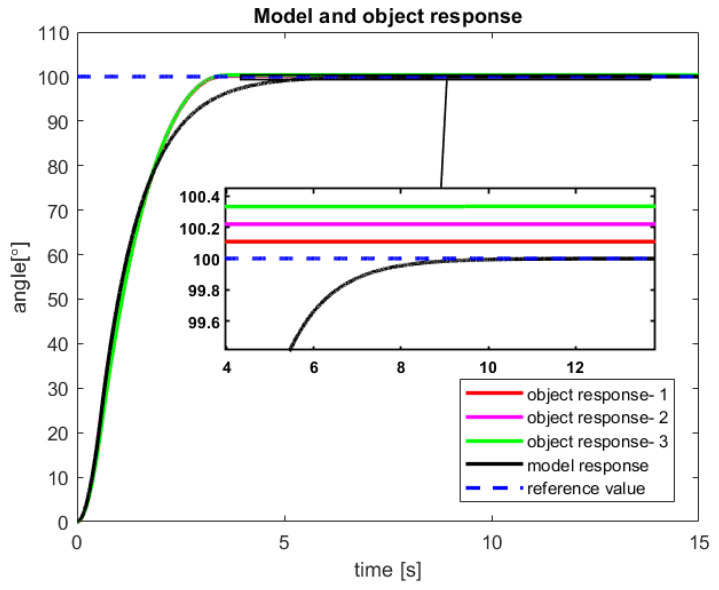
The received angular position of the model and the sensor-aided object—results of the three attempts with 100° reference value (source: authors).

**Figure 11:**
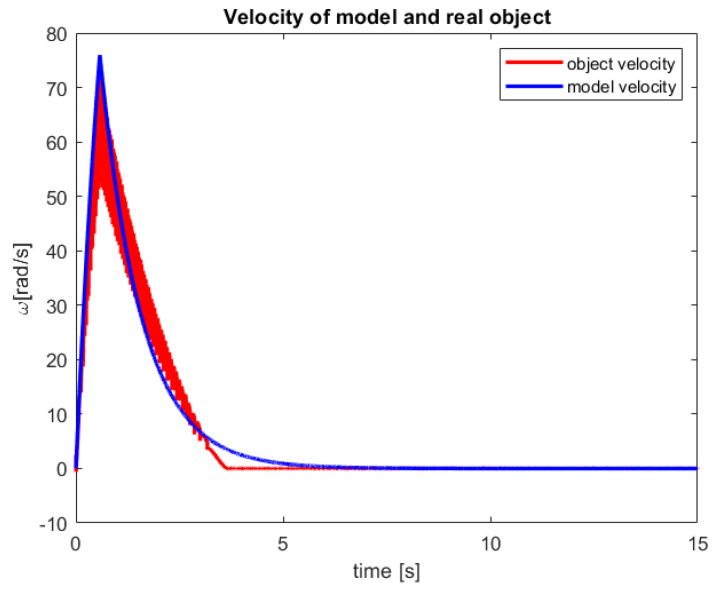
The received velocity of the model and the sensor-aided object for 100° reference value (source: authors).

After a verification of the control process of the sensor-aided system, the received responses of the model and real-life object have been presented in Figure 10 and Figure 11. The first one includes graphs that show the obtained servo angle positions both from the model and three sample attempts to control of the object. Subsequently, the second one depicts the received angular velocity.

The control process is characterized by a minimal inaccuracy in controlling the real object (Figure 10). This phenomenon is probably caused by factors such as measuring error of the encoder, play on the motor shaft (object wear), or inaccuracy in the modeling process. Nevertheless, a steady-state error of less than 0,4% is rather good accuracy for the first tests of the new introduced control law. Moreover, from the presented Figure 10, it can be seen that for all three trials that the real object reached the reference value much faster than the model.

In Figure 11, a very fast increase in the angular velocity value can be noticed. This phenomenon is natural for CTPC and now for new established RCTPC. Furthermore, taking into account the characteristics of the presented control, it can be seen that the model has a smoother course than the sensor-aided system, which is probably caused by the resolution of the encoder.

In the next subsection, an experiment with the new control law has been performed for another operating point.

### 8.2. The Second Experiment with the RCTPC Law

The second experiment with the new Real Continuous-Time Perfect Control has been performed for the reference angle value of 300°. The object linearization has been executed in the same manner such as in the previous Section 8.1. In the examined case, the received gain value was $K_{s}=185$, while the parameter $T_{s}$ remained unchanged. Therefore, for the considered system, the appropriate angular velocity and angular position transfer-functions take the following form:

(39) $$G_{\omega }\left(s\right)=\frac{185}{1.04s+1}\;\;\;\mathrm{and}\;\;\;G_{\alpha }\left(s\right)=\frac{185}{s(1.04s+1)}.$$

Meanwhile, the matrices in the state-space representation are as follows:

(40) $$A=\left[\begin{array}{cc} 0 & 1\\ 0 & -0.96 \end{array}\right],\;B=\left[\begin{array}{c} 0\\ 14.82 \end{array}\right],\;C=\left[\begin{array}{cc} 1 & 0\\ 0 & 1 \end{array}\right].$$

**Figure 12:**
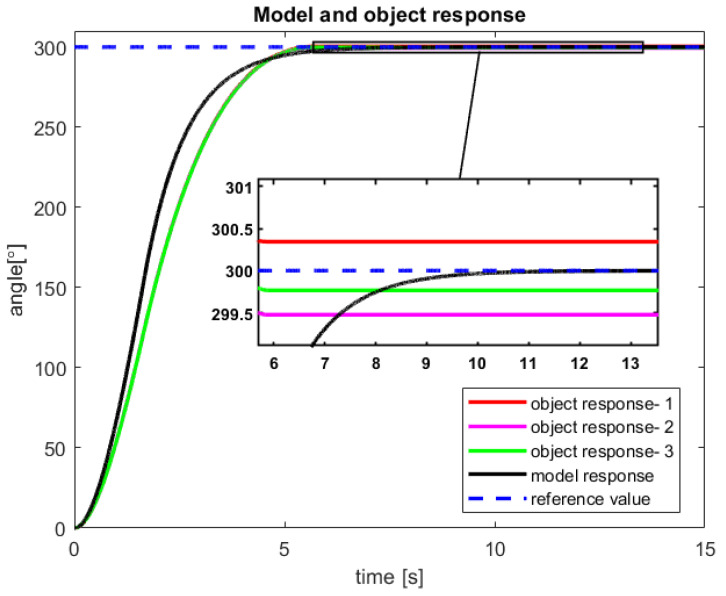
The received angular position of the model and the sensor-aided object—results of the three attempts with 300° reference value (source: authors).

**Figure 13:**
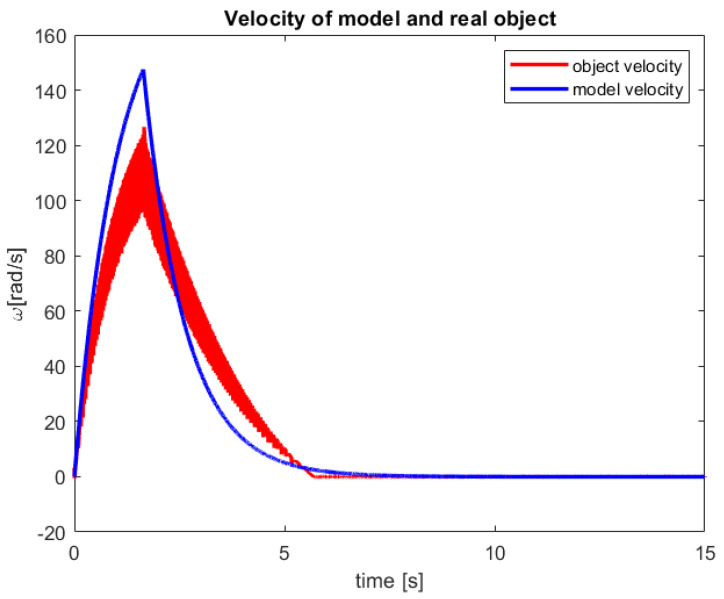
The received velocity of the model and the sensor-aided object for 300° reference value (source: authors).

After studies are performed on a sensor-aided system, the received results of the RCTPC algorithm have been presented in Figure 12 and Figure 13. The accomplished verification confirms the previously presented advantages and disadvantages of the new control law dedicated to the real-life objects.

In the future research effort, the control structure form Figure 9 should be slightly changed to receive feedback from the real-life object as well as the model. This approach should erase the revealing steady error of control process.

To confirm the usefulness of the new RCTPC algorithm, the classical PID control is presented in the next section. This comparison should clarify any doubts related to the advantages of the new perfect control dedicated to the real-life objects.

### 8.3. Experiment with PID Regulator

**Figure 14:**
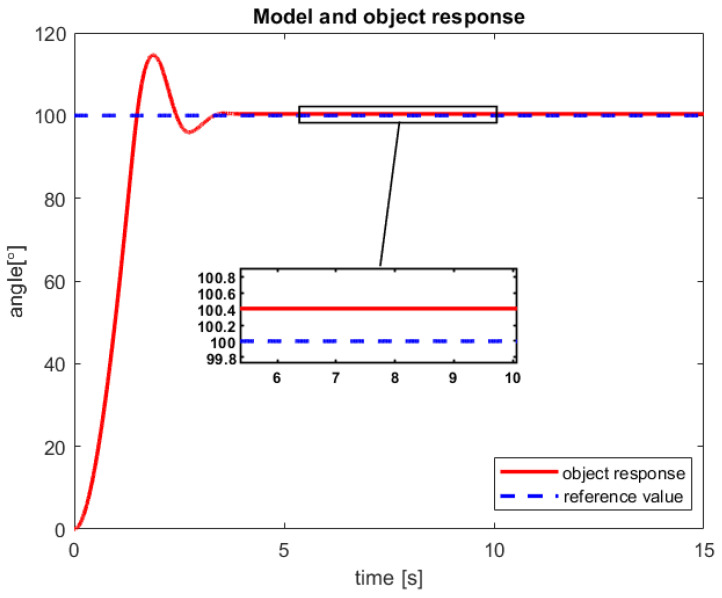
The received angular position of the the sensor-aided object for 100° reference value (source: authors).

**Figure 15:**
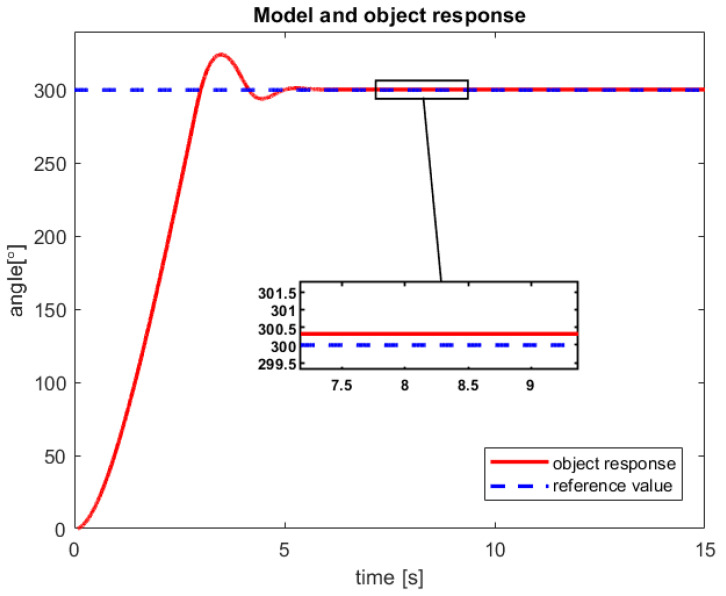
The received angular position of the the sensor-aided object for 300° reference value (source: authors).

To compare the research performed in the previous two subsections, the PID regulator has been selected. This approach has been chosen due to the fact that this type of control has been provided by the manufacturer of the considered servomechanism real-life object. Therefore, for each reference angle considered in previous studies, one test with the PID regulator has been performed. The system has been controlled according to Figure 9, which means that this control is performed in the same manner as in the case of the perfect control algorithm, which enables the research to be unambiguous. The received results for the reference values 100° and 300° have been depicted in Figure 14 and Figure 15, respectively. The mentioned graphs are intended to provide a comparative analysis between the new perfect control versus the control dedicated by the manufacturer. Thus, it was decided to perform only one test for each of the two reference values of the angular position.

The received results presented in whole Section 8 should carefully be reviewed in terms of performance indices. Therefore, the next paragraph of the article will provide an effective overview and discussion of the achieved outcome.

## 9. Discussion on the Obtained Sensor-Aided System Control Results

**Figure 16:**
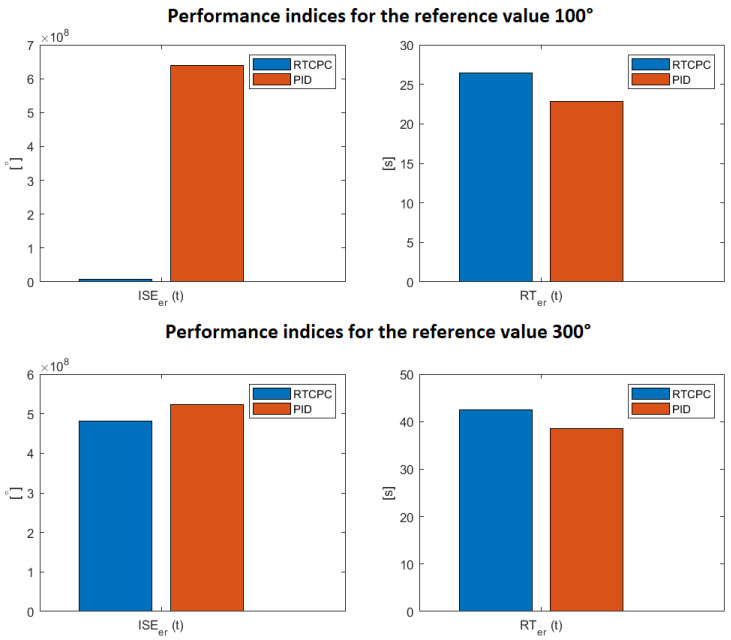
Graphical presentation of Table 4 (source: authors).

The results obtained during the research studies have been subjected to the performance indices. Moreover, the used ISE quality index has been considered in three different configurations. In the first one, the error between the real-life object (signal from the sensor) and the reference value has been measured. In the second one, the error of the model control, whilst in third one, the difference between model and sensor-aided system has been taken into account. These approaches make it possible to receive three types of analyzed errors $e_{r}\left(t\right)$, $e_{m}\left(t\right)$, $e_{c}\left(t\right)$ for the mentioned structures. The results obtained on this basis are presented in Table 4, whereas their selected graphical instances are depicted in Figure 16.

The differences in the results of the quality criteria for the real object result from the factors mentioned in the previous section. Moreover, an increase in the ISE and RT errors is visible with an increase in the angle reference value. It is a natural phenomenon resulting from the integral nature of the revealed inaccuracies.

Comparing the perfect control system with a dedicated PID controller, a clear conclusion has been drawn. The RCTPC algorithm guarantees a better control of the angular position of the servo in terms of the ISE criterion. This is due to the lack of oscillations that could be observed in the PID-oriented control. Simultaneously, the RT performance index turned out to be better for the classical regulation. Hence, it can be seen that in the new introduced approach, the used safety margin can be reduced by increasing the value $y_{p.\mathrm{ref}}\left(t\right)$. This operation should shorten the control time while increasing the control energy consumption. However, as the example of the PID controller demonstrated, the aforementioned energy requirement is feasible in practice. Therefore, the discussed case warrants further research studies.

## 10. Conclusions and Open Problems

In this paper, the new perfect control law devoted to the continuous-time physical objects has been introduced. The presented approach guarantees an appropriate regulation of plants having high inertia and characterized by the control signal limitation property. In order to verify such a behavior, a set of the real-life-originated numerical tests has been conducted under the research studies. Moreover, this article constitutes a first attempt toward the implementation of the perfect control in the sensor-aided systems. Henceforth, a simple control structure, which successfully reduces the computational burden, can be effectively employed. Having the experience gained from the practical research, the key open problems have been formulated. Firstly, it would be interesting to examine the discussed perfect control algorithm in the context of the Luenberger state observer application. Secondly, in order to address a problem covering the transition between the continuous- and discrete-time plants resulting from some digital operations, a new perfect control law defined in the discrete-time domain should be invented. In the end, these challenges warrant further in-depth research investigations.

## Figures and Tables

**Table 1:** The results of the quality criteria for the cascade multi-tank system control.

	LQR	LQR−I	RCTPC
ISE (m)	2.431	1.843	1.638
RT (s)	$2.4\times 10^{2}$	$1.67\times 10^{2}$	$1.58\times 10^{2}$
**MOE** (m^3^/s)	$3.655\times 10^{-6}$	$4.999\times 10^{-6}$	$1.514\times 10^{-7}$

**Table 2:** The results of the quality criteria for the two-level thermal object control.

	LQR	LQR−I	RCTPC
ISE (°C)	$6.749\times 10^{4}$	$5.949\times 10^{4}$	$5.208\times 10^{4}$
RT (s)	$6.27\times 10^{2}$	$3.86\times 10^{2}$	$2.38\times 10^{2}$
MOE (W)	$7.784\times 10^{9}$	$8.877\times 10^{9}$	$9.599\times 10^{9}$

**Table 3:** The parameters of the sensor-aided servomechanism system [47].

Symbol	Description	Unit
v(t)	input voltage	V
i(t)	armature current	[A]
ω(t)	angular velocity of the rotor	[rad/s]
*R*	armature resistance	[Ω]
β	damping factor	-
$K_{e}\omega \left(t\right)$	electromagnetic field	-

**Table 4:** The results of the performance indices.

RCTPC	100 [°]	300 [°]
$\mathrm{ISE}\;e_{r}\left(t\right)$ [°]		
1test	$8.17\times 10^{6}$	$4.81\times 10^{8}$
2test	$8.15\times 10^{6}$	$4.77\times 10^{8}$
3test	$8.16\times 10^{6}$	$4.78\times 10^{8}$
$\mathrm{ISE}\;e_{m}\left(t\right)$ [°]		
1test	$7.76\times 10^{6}$	$4.93\times 10^{8}$
2test	$7.76\times 10^{6}$	$4.93\times 10^{8}$
3test	$7.76\times 10^{6}$	$4.93\times 10^{8}$
$\mathrm{ISE}\;e_{c}\left(t\right)$ [°]		
1test	$5.12\times 10^{4}$	$2.31\times 10^{6}$
2test	$5.36\times 10^{4}$	$2.32\times 10^{6}$
3test	$5.57\times 10^{4}$	$2.35\times 10^{6}$
$\mathrm{RT}\;e_{r}\left(t\right)\;$[s]		
1test	$2.65\times 10^{1}$	$4.25\times 10^{1}$
2test	$2.64\times 10^{1}$	$4.27\times 10^{1}$
3test	$2.64\times 10^{1}$	$4.28\times 10^{1}$
$\mathrm{RT}\;e_{m}\left(t\right)\;$[s]		
1test	$3.3\times 10^{1}$	$3.93\times 10^{1}$
2test	$3.3\times 10^{1}$	$3.93\times 10^{1}$
3test	$3.3\times 10^{1}$	$3.93\times 10^{1}$
**PID**	**100** [°]	**300** [°]
$\mathrm{ISE}\;e_{r}\left(t\right)$ [°]		
PIDtest	$6.4\times 10^{8}$	$5.24\times 10^{8}$
$\mathrm{RT}\;e_{r}\left(t\right)\;$[s]		
PIDtest	$2.28\times 10^{1}$	$3.85\times 10^{1}$

## Data Availability

Data are contained within the article.

## Appendix A

In the Table A1 and Table A2, the parameters used during the simulation studies have been presented. The first table contains the parameters of the multi-tank system, while the second one applies the criteria operating in the two-level thermal object.

**Table A1:** The cascade multi-tank parameters.

Symbol	Description	Value	Unit
$H_{1max}$	height of the first tank	0.35	m
$H_{2max}$	height of the second tank	0.35	m
$H_{3max}$	height of the third tank	0.35	m
$C_{1}$	cross-section of the first valve	$1.0057\;\cdot \;10^{-4}$	m^2^
$C_{2}$	cross-section of the second valve	$1.1963\;\cdot \;10^{-4}$	m^2^
$C_{3}$	cross-section of the third valve	$9.8008\;\cdot \;10^{-4}$	m^2^
$H_{10}$	initial liquid height of the 1st tank	0.12	m
$H_{20}$	initial liquid height of the 2nd tank	0.8	m
$H_{30}$	initial liquid height of the 3rd tank	0.15	m
$\alpha_{1}$	flow factor of the first tank	0.5	-
$\alpha_{2}$	flow factor of the second tank	0.5	-
$\alpha_{3}$	flow factor of the third tank	0.5	-
*R*	radius of the third tank	0.365	m
*a*	the base of the first tank	0.25	m
*b*	distance between tanks	0.348	m
*c*	base of the second tank	0.1	m
*w*	the width of all tanks	0.035	m
$Q_{f}$	set flow through the pump	$9.8008\;\cdot \;10^{-5}$	(m^3^)/s
*q*	liquid inlet to the upper tank	-	(m^3^)/s
$q_{0}$	initial condition of liquid inlet	0.035	(m^3^)/s

**Table A2:** The two-level thermal object parameters.

Symbol	Description	Value	Unit
$T_{int}$	interior temperature	-	°C
$T_{int0}$	initial interior temperature	0	°C
$T_{ext}$	exterior temperature	-	°C
$T_{ext0}$	initial exterior temperature	−20	°C
$T_{att}$	attic temperature	-	°C
$T_{att0}$	initial attic temperature	0	°C
$Q_{h}$	heat power	-	W
$Q_{0}$	initial heat power	20,000	W
$C_{vin}$	interior thermal capacity	25,714.29	J/K
$C_{vat}$	attic thermal capacity	5714.29	J/K
$K_{ae}$	loss coefficient of the roof	60	-
$K_{ie}$	loss coefficient of the external walls	80	-
$K_{ia}$	loss coefficient of the ceiling	50	-
